# Identification of surgically-induced longitudinal lesions of the equine deep digital flexor tendon in the digital flexor tendon sheath using contrast-enhanced ultrasonography: an *ex-vivo* pilot study

**DOI:** 10.1186/s13028-014-0078-7

**Published:** 2014-11-25

**Authors:** Andrea Bertuglia, Giulia Mollo, Michela Bullone, Barbara Riccio

**Affiliations:** Dipartimento di Science Veterinarie, Università degli Studi di Torino, via Leonardo da Vinci 44, 10095 Grugliasco, TO Italy; Department of Clinical Sciences, Université de Montréal 3200 rue Sicotte, J2S2M2 Saint-Hyacinthe, QC Canada; Studio Veterinario Associato Cascina Gufa, strada provinciale 201 km 3, 26833 Merlino, LO Italy

**Keywords:** Microbubbles, Sulfur-hexafluoride, Contrast ultrasonography, Angle contrast ultrasonography, Synovial sheath, Intrathecal injection, Cadaver limb, Horses

## Abstract

**Background:**

Longitudinal tears in the lateral aspect of the deep digital flexor tendon are the most common causes of pain localised to the equine digital flexor tendon sheath. However conventional ultrasonographic techniques provide limited information about acute lesions. Ultrasonographic contrast agents are newly developed materials that have contributed to advancement in human diagnostic imaging. They are currently approved for intravenous use in human and animal models. In this study we described intrathecal use in the horse. This study was undertaken to evaluate the reliability of standard and angle contrast-enhanced ultrasonography to detect and characterize surgically-induced longitudinal lesions in the deep digital flexor tendons.

In this pilot study surgically-induced lesions were created in the lateral aspect of the deep digital flexor tendon within the digital flexor tendon sheath in 10 isolated equine limbs to generate a replicable model for naturally occurring lesions. Another 10 specimens were sham operated. All the limbs were examined ultrasonographically before and shortly after the intrasynovial injection of an ultrasound contrast agent containing stabilised microbubbles. The images were blindly evaluated to detect the ability to identify surgically-created lesions. The deep digital flexor tendons were dissected and a series of slices were obtained. The depth of longitudinal defects identified with contrast-enhanced ultrasound scans was compared to the real extent of the lesions measured in the corresponding gross tendon sections.

**Results:**

Contrast-enhanced ultrasonography with both angle and standard approach provided a significant higher proportion of correct diagnoses compared to standard and angle contrast ultrasonography (p < 0.01). Contrast-enhanced ultrasonography reliably estimated the depth of surgically-induced longitudinal lesions in the deep digital flexor tendons.

**Conclusion:**

Contrast-enhanced ultrasound of the digital flexor tendon sheath could be an effective tool to detect intrasynovial longitudinal tears of the deep digital flexor tendon, although an *in vivo* study is required to confirm these results for naturally occurring lesions.

## Background

Longitudinal tears (LTs) of deep digital flexor tendon (DDFT) are the most commonly recognized cause of digital flexor tendon sheath tenosynovitis and lameness localized to the digital flexor tendon sheath in horses [1]. The most reliable technique available to confirm the presence of LTs is surgical tenoscopy of the digital flexor tendons sheath [2]. Also magnetic resonance (MR) imaging did successfully identify lesions of the DDFT in its more distal aspect [3]. However, both of these techniques require general anaesthesia. Clinically, the diagnostic approach to DDFT’s lesions more commonly consists in ultrasound (US) examination. It easily allows detecting non-specific signs of acute and chronic tenosynovitis [4], such as echogenic synovial proliferations and irregularities of the DDFT borders, which are findings highly predictive of LTs [5,6].

The detection of LTs in their acute stage is challenging. In two case series, the percentage of LTs identified at ultrasonography varied from 49% [6] to 76% [5] compared to surgical findings, and this difference was most likely related to the higher definition of the US machines and the growing experience of the operators, so that the current accuracy of detecting these lesions is probably still increased. Furthermore, the fibres of the tendon are stretched longitudinally during weight bearing, which accentuates their juxtaposition and prevents the identification of existing longitudinal defects. In these cases, only an US examination of the digital flexor tendon sheath with the fetlock joint maintained in a semi-flexed position would allow visualising the lesion. Alternative imaging approaches have been described which could enhance visualization of the LTs of the DDFT. During angled ultrasound, the probe is placed laterally to the palmar/plantar surface of the limb. It yields oblique and transverse images of the tendon and improves the detection of LTs at the DDFT margin, but their diagnosis remains subjective [5,6]. Angle contrast US (ACUS) can be obtained by angling the probe in such a way to achieve an oblique incidence image of the tissues. Angle contrast US enhances the marginal contrast of the tendon and provides additional diagnostic information compared to standard US on the proximal aspect of the equine suspensory ligament [7]. However, no studies have investigated its effectiveness in diagnosing LTs of the DDFT. Intrathecal contrast radiography, performed concurrently with diagnostic analgesia, has been proposed as an alternative imaging modality to identify DDFT tears, with an overall sensitivity of 57% and a specificity of 84% [8].

MR imaging is often used to diagnose soft tissue lesions that are difficult to identify at US. MR imaging is superior to US for detecting changes in the tissue fluid content, and it is considered the gold standard diagnostic technique for DDFT injuries localized into the hoof capsule [9,10]. However, LTs of the DDFT into the digital flexor tendon sheath have been poorly characterized in horses [11]. Clearly, improving the imaging techniques used when lesions within the equine digital sheath are suspected provides precious additional diagnostic information.

Contrast-enhanced US (CEUS) employs injectable highly-echogenic stabilized microbubbles that increase the ultrasound backscatter producing highly contrasted sonograms. Such microbubbles are made of sulfur hexafluoride with an external phospholipid shell, and have a mean diameter of 2.5 μm (range 0.7–10 μm) [12]. Although CEUS has been generally employed for the investigation of tissue vascularity and perfusion [13], its use in musculoskeletal imaging has been increasing. In the last years, CEUS has been successfully used in man for diagnosing rotator cuff lesions of the shoulder [14] as well as synovitis in patients with knee osteoarthritis [15] and inflamed sacroiliac joints [16].

We hypothesized that CEUS improves the detection of LTs of the DDFT in an *ex-vivo* model of the lesion in its acute phase. Our primary objective was to investigate whether the use of a contrast agent increased the sensitivity of two different US approaches (standard and angled) in detecting intrasynovial surgically-induced longitudinal lesions of the DDFT. Also, we studied the reliability of CEUS in determining the extent of the lesions.

## Material and methods

### Specimens

Twenty thoracic and pelvic limbs of skeletally mature horses with unknown lameness history were collected at the slaughterhouse shortly after euthanasia.

### Study design

Ten limbs were randomly assigned to the operated group. Surgically-induced longitudinal lesions were created in the intrasynovial portion of the DDFTs along the lateral margin of the tendons, using the technique described below. The remaining 10 specimens were assigned to the sham-operated group, in which only two skin incisions were made and sutured in the same position as in the first group for unbiased analysis. All specimens were scanned before and immediately after intrathecal injection of a commercially available ultrasonography contrast medium with stabilized microbubbles^a^. An experienced observer evaluated the presence or the absence of a surgically-induced longitudinal lesion in the intratechal portion of the DDFT blindly. Deep digital flexor tendons were then carefully dissected from the specimens and processed for morphometric analysis.

### Experimental surgery

**Figure 1:**
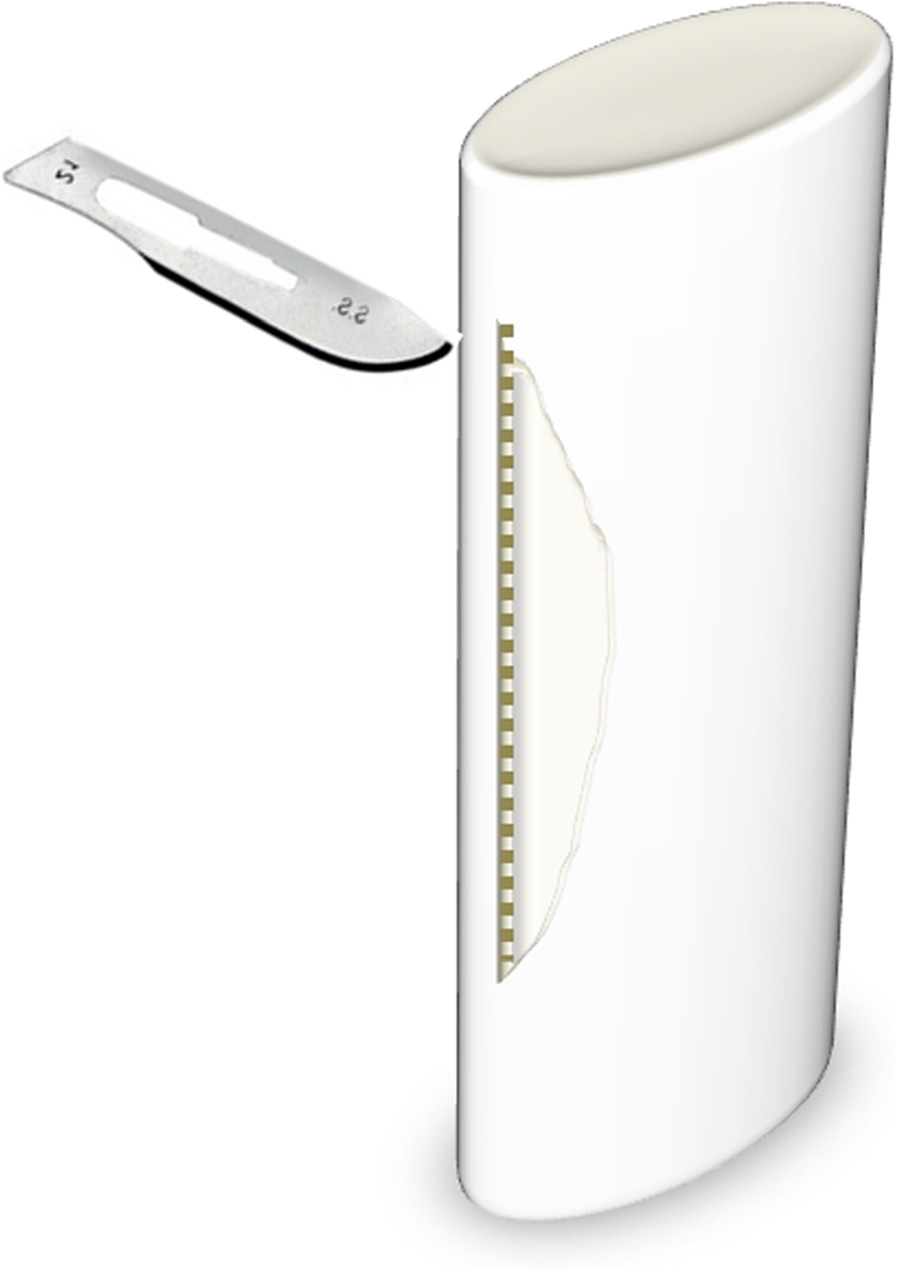
**Schematic representation of the method to create intratendonous stab incisions in the equine deep digital flexor tendons.**

The limbs were prepared as for a standard tenoscopy and a surgically-induced longitudinal lesion was created along the lateral aspect of the DDFTs under endoscopic guidance. The specimens were kept immersed in water the entire time of the procedure, in order to prevent air trapping within the soft tissues (producing artefacts at the following US examination). An intra-tendonous stab incision was performed along the intrasynovial portion of the DDFT using a N.11 scalpel blade, kept parallel to the tendon fibres as shown in Figure 1. The metacarpo(tarso)phalangeal joint was maintained with a dorsal angle of 180° during the procedures. A double-portal endoscopic technique was employed. A 30°, 4,5-mm Ø, 18-cm long rigid arthroscope^b^ was positioned through the first arthroscopic portal, made at the proximal and lateral pouch of the fluid-distended digital flexor tendon sheath. The second operative arthroscopic portal was made approximately 5 cm-distally to the first one, along to the lateral aspect of the DDFT, proximally to the palmar annular ligament. The same operator performed all the procedures. An effort was made to mimic the extent and position of naturally occurring lesions. The cutaneous incisions were sutured with a single mattress suture using N.0 Ø nylon on a reverse cutting needle.

### Standard and angle contrast ultrasound

Ultrasonographic images were obtained using a portable ultrasound machine^c^ with a 10 MHz linear probe and a standoff pad. During ultrasonographic examination, specimens were maintained vertically by means of a customized adjustable press simulating the weight bearing position. The same experienced operator examined all the specimens. Transverse images were obtained approximately at one centimetre-distance along the digital flexor tendon sheath, beginning at its proximal part and down to the proximal sesamoid bones. For each section a standard US scan and an angle scan (ACUS, angling the probe approximately at 10°) were performed, and the images were digitally stored.

### Microbubbles injection

A commercially available sulphur hexafluoride microbubbles solution was employed as ultrasonographic contrast medium. The solution was prepared according to the manufacturer’s instructions, reconstituting 25 mg of lyophilised powder into a volume of 10 ml sterile saline, in order to reach a concentration of 2–3 × 10^8^ microbubbles/ml. This solution was injected intrathecally using a 19G needle, with a basilar sesamoidean approach to the digital flexor tendon sheath [17]. Withdrawing a small volume of synovial fluid before the injection of the contrast medium confirmed the correct placement of the needle. The fetlock was then flexed and extended repeatedly for 1 minute, in order to allow the contrast medium distributing homogeneously into the synovial space.

### Contrast-enhanced ultrasound and angle contrast-enhanced ultrasound

The specimens were positioned as described for the standard US and a complete series of transverse images were obtained with the same technique used before by the same operator. Contrast-enhanced ultrasound and angle contrast-enhanced US (ACEUS) scans were obtained and the images were stored for subsequent analysis.

### Morphometric study

Deep digital flexor tendons included in the study were completely and carefully harvested from the specimens studied. Consecutive 1-mm thick transverse sections were obtained from each DDFT (including the region where the longitudinal incisions were macroscopically detectable), numbered in a progressive order from proximal to distal, and frozen. Digital images including a centimetre-scale reference were obtained for each section. A single operator performed all the morphometric evaluations blindly using a Java-based image processing program^d^. The software measurement scale was set for each specimen. The depth of the longitudinal defects was computed for each transverse tendon’s tissue section. The US images were analysed using the same protocol. The transverse extent of the surgically-induced longitudinal lesions was determined by consensus with the ultrasonographer.

### Statistical analysis

Statistical analyses were performed using Prism 6.0 software^e^ and online at http://scistatcalc.blogspot.co.uk. The proportion of correct and incorrect assessments was calculated for standard US, ACUS, CEUS and ACEUS in the sham and experimentally operated limbs. Dissected tissues were our reference. The percentages of correct and incorrect diagnoses obtained with the four different US techniques (standard US, ACUS, CEUS, ACEUS) were compared using a Cochran’s Q test followed by a post-hoc analysis and a pairwise McNemar test for non-independent variables. Interclass correlation coefficient (ICC) and a Bland-Altman analysis were performed to assess the agreement between the measurements of the surgically-induced lesion depth at gross pathology and ultrasound (ACEUS scans). The measurements carried out on transverse tissue’s sections were used as a reference for the Bland-Altman test. The level of statistical significance was set at p < 0.05.

## Results

### Descriptive data

**Figure 2:**
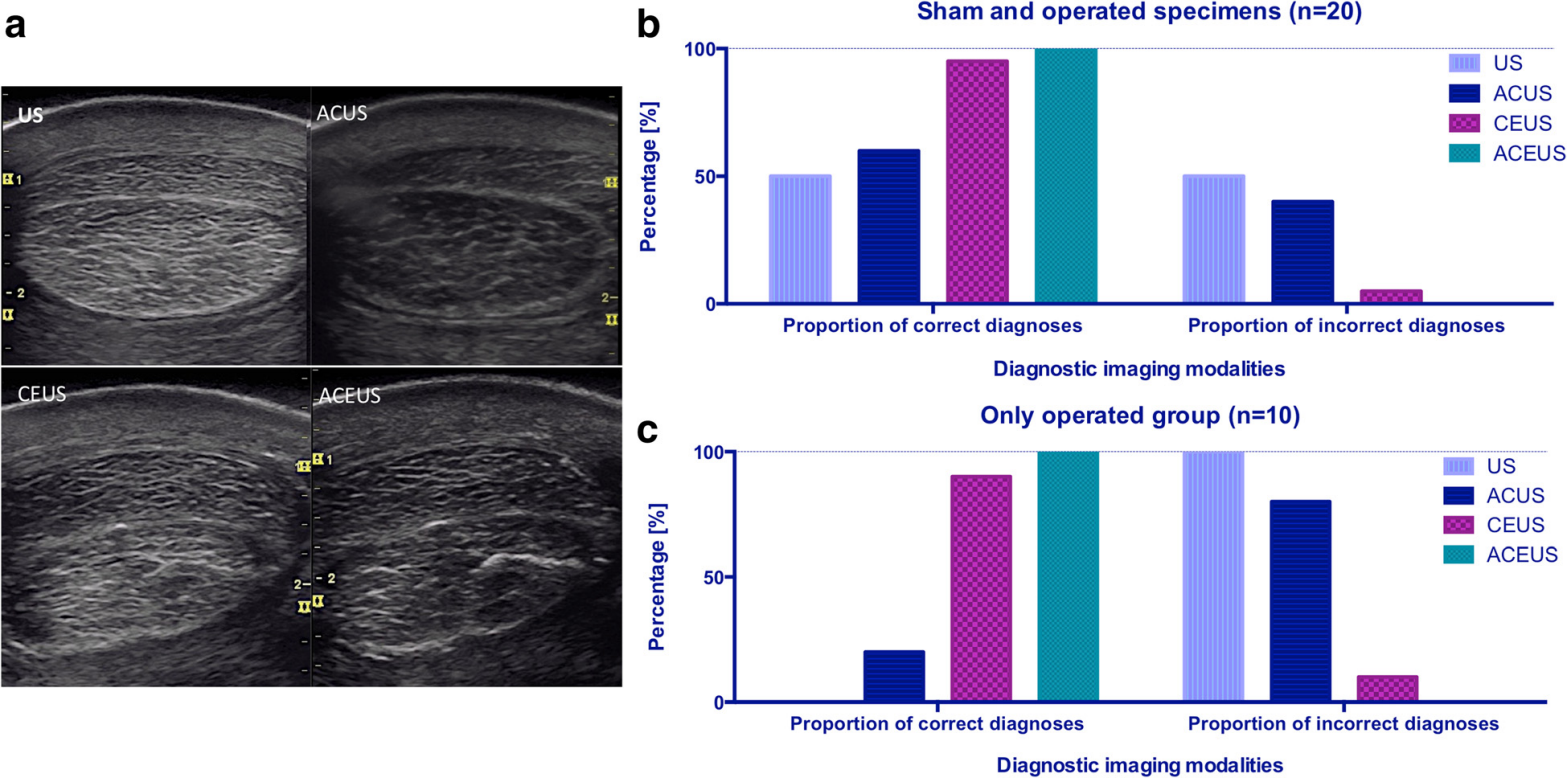
**Ultrasonographic techniques and proportion of correct and incorrect diagnoses.** In the panel **(a)** four ultrasonographic techniques are shown in the same speciment to identify surgical induced longitudinal lesion at the lateral margin of the deep digital flexor tendon. In the upper line of the panel, the intrasynovial portion of the tendon is visualized at standard US and at ACUS, before the injection of microbubbles contrast medium. In the line below the corresponding CEUS and the ACEUS are shown. **(b)** The proportion of correct and incorrect diagnoses of artificial tears are reported in the group of sham and operated specimens **(b)**, and in the group of the operated limbs alone **(c)**. In the group in **(b)** the proportion of correct diagnoses using ACUS and US is around 50%. This resulted from the poor ability to identify surgical induced longitudinal lesions in the operated limbs **(c)**, where the proportion of incorrect diagnoses with standard US is 100%. Abb: CEUS = constrast enhanced ultrasound; ACEUS = angle contrast enhanced ultrasound; ACUS = angle contrast ultrasound; US = ultrasound.

Contrast medium intratechal injection resulted in a diffuse hyperechoic appearance of the synovial fluid into the equine digital flexor tendon sheath, with a non-uniform distribution around the flexor tendons. Surgically-induced longitudinal lesions of the DDFT were identifiable as hyperechoic lines within the DDFT’s structure, extending from its lateral margin towards the tendon cores (Figure 2). Air artefacts were not detected into the digital flexor tendon sheaths during the US examination of the operated limbs, which confirmed the effectiveness of the surgical technique used to generate longitudinal incisions in our experimental model.

### Comparison between ultrasonographic imaging modalities

Results of the US scans using the four different techniques to discriminate surgically-induced longitudinal lesions are summarized in Figure 2. Standard US and ACUS approaches, in the absence of contrast medium, permitted the diagnosis of 0% and 20% of surgically-induced longitudinal lesions, respectively. Contrarily, CEUS effectively identified 90% of longitudinal incisions, whereas ACEUS outlined 100% of the lesions in our experimental model. In two cases, the hyperechoic lines within the tendon structure appeared as interrupted when the images were acquired with the standard CEUS, whereas ACEUS allowed the complete visualization of the lesions. The percentage of correct assessment of the lesion (by considering only its presence or absence) was significantly higher when CEUS and ACEUS were employed compared to standard US (p = 0.002 and p = 0.001, respectively) and to ACUS (p = 0.008 and p = 0.004, respectively). Standard US and ACUS approaches yielded similar percentage of correct assessment of the lesion (p = 0.1), such as CEUS and ACEUS (p = 0.3). False positive lesions were not diagnosed when CEUS and ACEUS were used, indicating that microbubbles injection *per se* does not produce significant artefacts leading to misinterpretation of the images.

### Comparison of morphometric findings with contrast enhanced US scans

**Figure 3:**
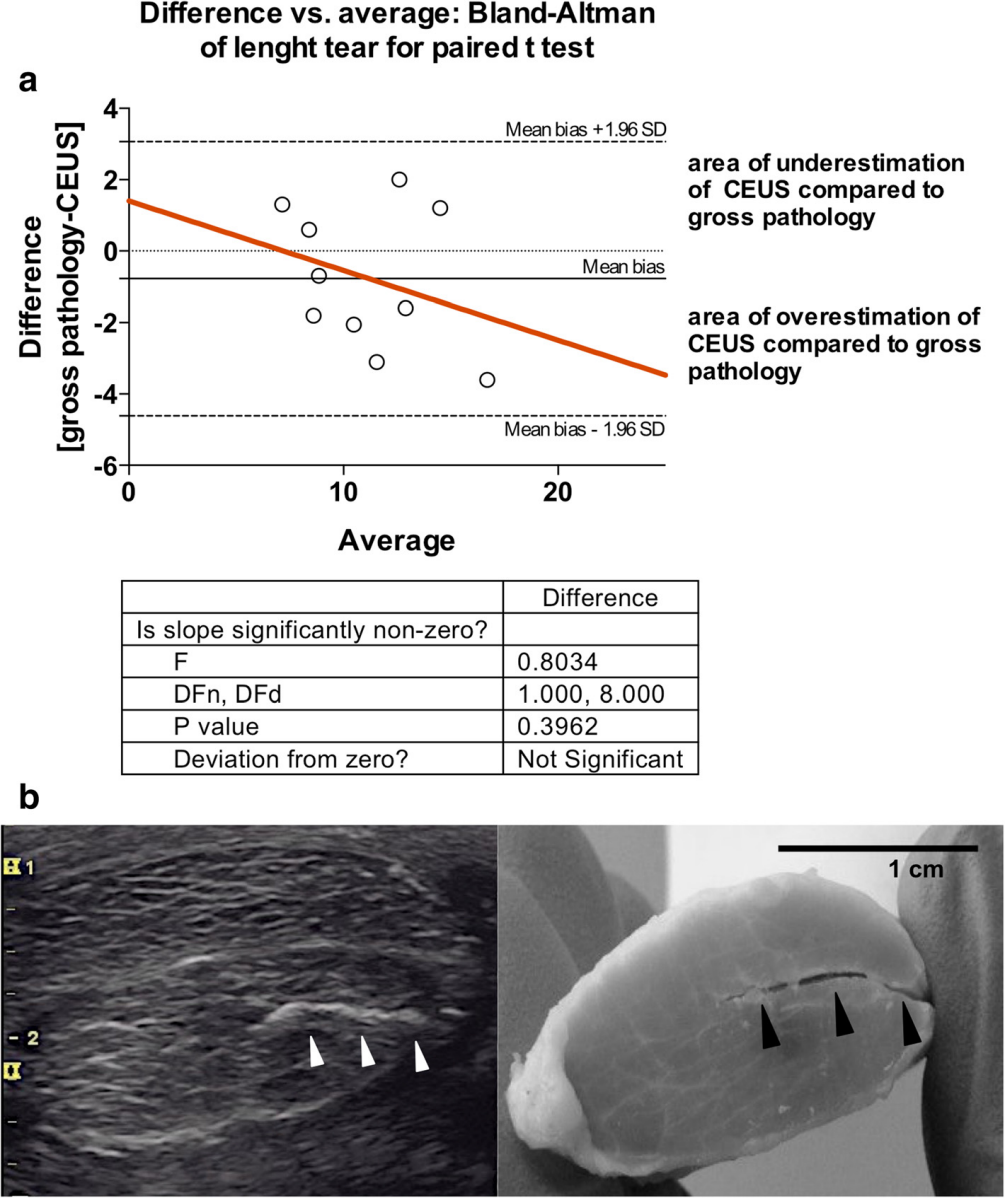
**Comparison between measurements of the surgical incisions depth at contrast-enhanced ultrasound scans and in corresponding tissue sections. (a)** The Bland-Altman difference plot (n = 10) indicates the correlation between measurements at angle contrast-enhanced US scans and gross pathology. The difference is plotted against mean value, and the 95% limits of agreement (mean bias ± 1.96 SD) of the difference between the two methods of measurement are shown, as is the regression line (red). **(b)** The angle contrast enhanced US scan and the corresponding tissue sections are reported. The hyperechoic line (white arrows heads) and the corresponding lesion in the transverse section of the dissected tendon (black arrows heads) are shown. Abb: US = ultrasound; SD = standard deviation.

At gross pathology, the mean longitudinal extent of the surgically-induced longitudinal lesions was 9.8 ± 2.2 mm (mean ± SD), while their mean depth was 11.1 ± 2.3 mm. The depth of the incisions was calculated for each specimen as the mean of all the measures made on the transverse sections, in which the lesion was detectable. The ICC for the measurements made at gross pathology and US was 0.89 indicating that the level of concordance was high. The Bland-Altman test indicated that contrast-enhanced ultrasound reliably estimated the experimental incisions depth with a mean bias of 0.77 ± 1.99 mm (Figure 3). As the slope of the Bland-Altman curve was not significantly different from 0 (p = 0.4, F test), the transverse extent of the surgical incision did not represent a source of systematic significant bias at US interpretation.

## Discussion

This work aimed at investigating the reliability of CEUS compared to the standard and angle US approaches in detecting and evaluating the extension of artificially induced lesions of the intrasynovial portion of the equine DDFT. We used an *ex-vivo* model in which the lesions were surgically performed and US examinations were made before and after the injection of a contrast medium into the equine digital flexor tendon sheath. Standard and angle US approaches were repeated in presence and absence of contrast medium. Contrast-enhanced US techniques (both standard and angled) significantly increased the marginal contrast of the anatomical structures within the equine digital flexor tendon sheath in our model, allowing ultrasonographic identification of surgically-induced longitudinal lesions in a significantly higher proportion of cases compared to standard and angle US. Also, CEUS provided an accurate estimate of the real depth of the lesions created into the tendon structure. Overall, our results support a role for CEUS in *in vivo* diagnosis of acute intrasynovial lesions of DDFTs, when chronic changes are not evident yet detectable.

The first clinical implication of our investigation was to determine whether the CEUS could have a role in practice to diagnose LTs of DDFT in their acute stage. Indeed, during chronic tenosynovitis, the digital flexor tendon sheath effusion and synovial proliferations induced by the protracted inflammatory process can assist the clinician in identifying this type of lesion [4-6]. In our model of acute damage, surgically-induced longitudinal lesions in the DDFT could not be identified by standard US. However, the contrast medium easily diluted within the synovial fluid and into the lesions, delineating surprisingly well their extension and producing a hyperechoic lines that strongly contrasted with the echogenicity of the tendon structure.

The angle contrast technique further increased this contrast by reducing the echogenicity of the collagen fibres, as microbubbles echogenicity is not angle-dependent [7]. The angle contrast approach in presence of the contrast medium (ACEUS) allowed correct identification of experimental lesions in all the cadaveric limbs studied, but did not yield remarkable advantages in diagnosing surgically-induced longitudinal lesions compared to the standard CEUS approach.

Lesions that we created in the tendons were similar in depth to naturally occurring injuries extending approximately 10 mm [5,6]. However, longitudinal extent of the lesions was reduced in our experimental model compared to spontaneous cases, which has been reported to extend up to 7 cm in some clinical cases [5]. It is possible that as our lesions were shorter than in real cases, their identification was more difficult in our experiment compared to naturally occurring injuries. From another point of view, the presence of increased concentrations of acute phase proteins within the synovial space could alter the diffusing capacity of the contrast medium into the tear *in vivo*, negatively affecting the diagnostic accuracy of the CEUS technique.

Microbubble contrast medium is typically employed intravascularly in human orthopaedics, in order to enhance blood contrast among different tissues [18]. In our study we have injected the microbubbles directly into the synovial space. While several clinical investigations reported a low incidence of adverse effect following intravascular administration of sulphur hexafluoride microbubbles [19], intrasynovial tolerance is poorly investigated. Microbubbles are isotonic and are claimed to be devoid of antigenic potential [20]. Their intrasynovial tolerance was evaluated in rabbit knees. The procedure was found to be safe with minimal and transitory histological changes of the synovial membrane [21]. The dose of microbubble dispersion used to highlight surgically-induced longitudinal lesions in our study was identified with unpublished experiments previously performed in isolated equine limbs. The volume of the contrast medium we employed in this study was satisfactory for diagnostic purposes *ex-vivo*.

We acknowledge that our experimental method lacks important features of disease chronicity helping in the US diagnosis of LTs of the DDFT, such as synovial proliferations and granulomata, as well as irregularities of DDFT’s margins and/or digital sheath effusion. The limbs we studied were not subjected to cyclical loading, which could possibly separate the edges of the tear and helping identifying naturally occurring lesions. Also, the fact that synovial proliferations could represent an obstacle to the penetration of the contrast medium into a spontaneous LT is noteworthy. For all this reasons, an *in vivo* validation of our technique is needed before drawing conclusion about its possible clinical application. We anticipated that an important concern for *in vivo* intratechal CEUS application would be related to avoid any outstretching of the digital flexor tendon sheath associated to the contrast medium injection, which could induce patient discomfort. Moreover, the decay time of microbubble’s contrast medium in the tissues should be investigated. *Ex-vivo*, in absence of vascular supply, it exceeded the time required to perform a complete US scan at the level of the digital flexor tendon sheath.

## Conclusion

Contrast enhanced ultrasound reliably identifies the presence and the depth of surgically-induced longitudinal lesions in the DDFT in our equine *ex-vivo* model. This technique represents a promising method for in field detection of LTs of the marginal aspect of the DDFT in their acute stage. Our results support the implementation of further investigation with the aim of validating the proposed technique *in vivo*.

## Endnotes

^a^SonoVue -Bracco, Milan, Italy.

^b^Dyonics, Smith & Nephew.

^c^Logiq E, General Electric Healthcare, Little Chalfont, UK.

^d^ImageJ, NIH Bethesda, MD, USA.

^e^GraphPad, La Jolla, CA, USA.

## Abbreviations

DDFT: Deep digital flexor tendon
US: Ultrasound
ACUS: Angle contrast ultrasound
CEUS: Contrast enhanced ultrasound
ACEUS: Angle contrast enhanced ultrasound
MR: Magnetic resonance
